# Advances in sex disparities for cancer immunotherapy: unveiling the dilemma of Yin and Yang

**DOI:** 10.1186/s13293-022-00469-5

**Published:** 2022-10-22

**Authors:** Junfu Ma, Yanxin Yao, Ye Tian, Kexin Chen, Ben Liu

**Affiliations:** 1 Department of Epidemiology and Biostatistics, Key Laboratory of Molecular Cancer Epidemiology, Tianjin, National Clinical Research Center for Cancer, Tianjin Medical University Cancer Institute and Hospital, Tianjin Medical University, Tianjin, 300060 China; 2 Department of Senior Ward, National Clinical Research Center for Cancer, Key Laboratory of Molecular Cancer Epidemiology of Tianjin, Tianjin Medical University Cancer Institute and Hospital, Tianjin, People’s Republic of China

**Keywords:** Sex disparities, Cancer immunotherapy, Immune checkpoint inhibitors (ICIs), Biomarker, Precision medicine

## Abstract

A wide sex disparity has been demonstrated in cancer incidence, tumor aggressiveness, prognosis, and treatment response of different types of cancer. The sex specificity of cancer appears to be a relevant issue in managing the disease, and studies investigating the role of sex and gender are becoming extremely urgent. Immunotherapy plays a leading role in cancer treatment, offering a new perspective on advanced malignancies. Gender has not been considered in standard cancer treatment, suggesting increasing the recognition of sex differences in cancer research and clinical management. This paper provides an overview of sex and gender disparities in cancer immunotherapy efficacy, anti-cancer immune response, predictive biomarkers, and so on. We focus on the molecular differences between male and female patients across a broad range of cancer types to arouse the attention and practice of clinicians and researchers in a sex perspective of new cancer treatment strategies.

## Highlights

- Sex differences exist in the prevalence, tumor invasiveness, treatment responses, and clinical outcomes of pan-cancer, suggesting that, while not yet incorporated, sex will probably be considered in future practice guidelines.
- Inherent genetic differences, overlapping epigenetic alterations, and sex hormone influences underpin everything. Androgen receptors influence the sexual differences in TME by regulating epigenetic and transcriptional differentiation programs. It highlights a sex-based therapeutic target for cancer immunotherapy.
- Proper consideration of sex, age, sex hormones/menopause status, and socio-cultural gender differences in clinical investigation and gene association studies of cancer are needed to fill current gaps and implement precision medicine for patients with cancer.

## Introduction

With the increasing global burden of cancer, it is worth noting that there are significant differences between men and women in terms of cancer incidence, tumor invasiveness, prognosis, and treatment responses to many types of cancer. It is well-known that non-reproductive system tumors show a biological solid sex difference, with high morbidity and mortality in males [1]. However, we still have a limited understanding of the molecular basis of sex disparities between male and female patients with cancer. Although the importance of gender and sex inclusion in oncology practice is increasingly recognized, its application remains blinded [2, 3].

Appropriate reporting of sex (male, female) and gender (man, woman) is vital in clinical research. For the most part, the sex of human subjects in biomedical research is known. "Sex" refers to the biological and physiological characteristics that define humans as male, female, or intersex [4]. "Gender", however, is a societal construct that refers to roles, activities, and behaviors and encompasses a wide range of identities beyond male, female, and intersex [5]. Defining gender in human studies is both difficult and controversial. Indeed, some have argued that sex and gender are "irreducibly entangled" and that even the most seemingly straightforward presentation of sex as a biological variable in human studies is inevitably a mix of sex and gender [6, 7]. The very concept of gender is subtle, complex, and shifting. Presently, the incorporation of gender variables within research remains limited [8], resulting in inadequate attention to the effect of gender-influenced behavior and exposures on health outcomes, which are separate from biological sex.

It is common for the two terms sex and gender to be used interchangeably. Both sex and gender affect molecular and cellular processes, clinical traits, treatment response, health, and disease [8]. Sex and gender work together to cause multiple sex differences in human clinical and pathology. Sex (i.e., the biological differences between men and women) and gender (i.e., behavioral differences associated with being male or female) are biological variables that can potentially affect immune responses to both foreign and self-antigens [9]. Sexual dimorphism of immunity could affect innate and adaptive immune responses and thus lead to functional diversity between different sexes, which has been largely ignored in immunotherapy so far [10]. Specific immunological sex differences exist throughout life, while other differences are only apparent after puberty and before reproductive senescence, indicating that genes and hormones are involved [9].

In humans, combining all sex-specific genetic, epigenetic, and hormonal influences of biological sex produces differences in vivo environments for male and female cells. However, apart from biological factors (sex), social behavior factors (gender) may also be attributed to the sex/gender disparity in immunity and cancer immunotherapy response. Sex and gender are potent covariates that are strongly associated [8]. Many determinants of disease, both physical and social, are differentially distributed by gender. For example, in many societies, women are more likely to be vitamin D deficiency [11], and men are more likely to smoke cigarettes and drink alcohol. Men and women are also exposed differentially to violence and trauma [12, 13]. Such stressors may affect gonadal steroid secretion in a sex- and hormone-dependent fashion [14].

The anti-cancer immune response of males is different from that of females. The difference is affected by the following factors: personal factors (e.g., cognitive/emotional), contextual factors, such as genetic mediators (e.g., sex chromosomes X/Y), hormonal mediators (e.g., estradiol, progesterone, and androgens), environmental mediators (e.g., the microbiome) [2], social sex behaviors (e.g., smoking and alcohol consumption) [15–17] and age factor [2]. Sex differences in cancer biology and treatment deserve more attention and systematic investigation. However, it has only been May 2014 that the US National Institutes of Health (NIH) announced that researchers should account for sex and consider it a biological variable (SABV) in NIH-funded preclinical research [18]. It is relevant that preclinical studies use animals of both sexes to investigate the molecular mechanisms underlying cancer development and progression. Furthermore, sex and gender should be considered in clinical trials for more accurate diagnosis, correct stratification of patients, and proper therapies [19]. In the era of precision medicine, the goal will be to identify molecular drivers, possibly different in males and females, to predict responders and non-responders and select the best therapeutic action for each.

Sex affects gene expression and its genetic regulation across tissues. Oliva et al. measured the effect of gender on gene expression in 44 human tissues from GTEx and integrated them with genotypes of 838 subjects. Their research showed that sex-biased gene expression is present in numerous biological pathways and is associated with sex-differentiated transcriptional regulation [20]. As expected, the most robust sex bias was observed for X-chromosome genes, whereas most sex-biased genes were autosomal, suggesting the influence of sex on genome-wide regulatory programs. In a word, sex and gender add to underlying genetic predisposition and influence of sex steroid hormones. These factors affect metabolism, immunity, inflammation, and ultimately the fidelity of the genetic code.

Typical male and female development diverge under the influence of sex and non-sex chromosomes, with determinant contribution by sex steroid hormones [20, 21]. Sex bias exists in the development and progression of non-reproductive organ cancers, but the underlying mechanisms are enigmatic. Kwon et al. established a role for CD8 + T cell-dependent anti-tumor immunity in mediating sex differences in tumor aggressiveness, which is driven by the gonadal androgens rather than sex chromosomes [22]. The capacity of sex hormones to influence the inception and progression of non-reproductive cancer has received a comprehensive review [23]. This mini-review summarizes recent research progress concerning sex differences in cancer immunotherapy. We aim to stimulate further research as the essential step in better developing cancer immunotherapy for personalized and patient-centered care. We cannot mention every study, as the literature is too vast to cover in complete detail. Therefore, we attempt to summarize only some of the more recent and salient observations in the field.

## Sex disparities in anti-cancer immunotherapy

Immune checkpoint blockade (ICB) therapies, including inhibition of programmed cell death 1 (PD-1) or ligand 1 (PD-L1) and cytotoxic T-lymphocyte antigen-4 (CTLA-4), have been able to induce a lasting response across multiple cancer lineages. However, the significant response of immunotherapy is currently limited to a small number of patients and indications [24]. It is well-established that there is a profound sex difference in immune responses, with females having a more active immune system than males [9], suggesting that the sexual dimorphism of the immune response plays a role in cancer pathogenesis.

Existing reports reveal the contradictory associations between ICIs benefits and sex in advanced cancers. Conforti et al. have shown that patients' gender influences the response to anti-cancer immunotherapy. When anti-CTLA4 or anti-PD-1 antibodies were used as monotherapy, men obtained more immense survival benefits in several solid tumors than women [25]. However, women with advanced non-small cell lung cancer (NSCLC) experienced an impressive, more considerable survival benefit than men from the combination of chemotherapy with an anti-PD-1 or anti-PD-L1 [26].

In addition, another meta-analysis showed no statistically significant difference in immunotherapy efficacy for overall survival between males and females [27]. It is important to note that different ICI agents may have other sex-based differences in their efficacy. For example, anti-CTLA-4 antibodies appear to have more significant sex differences than anti-PD-(L)1 antibodies [28, 29]. More shreds of evidence were needed to clarify the controversy of this sex-based discrepancy.

**Fig. 1:**
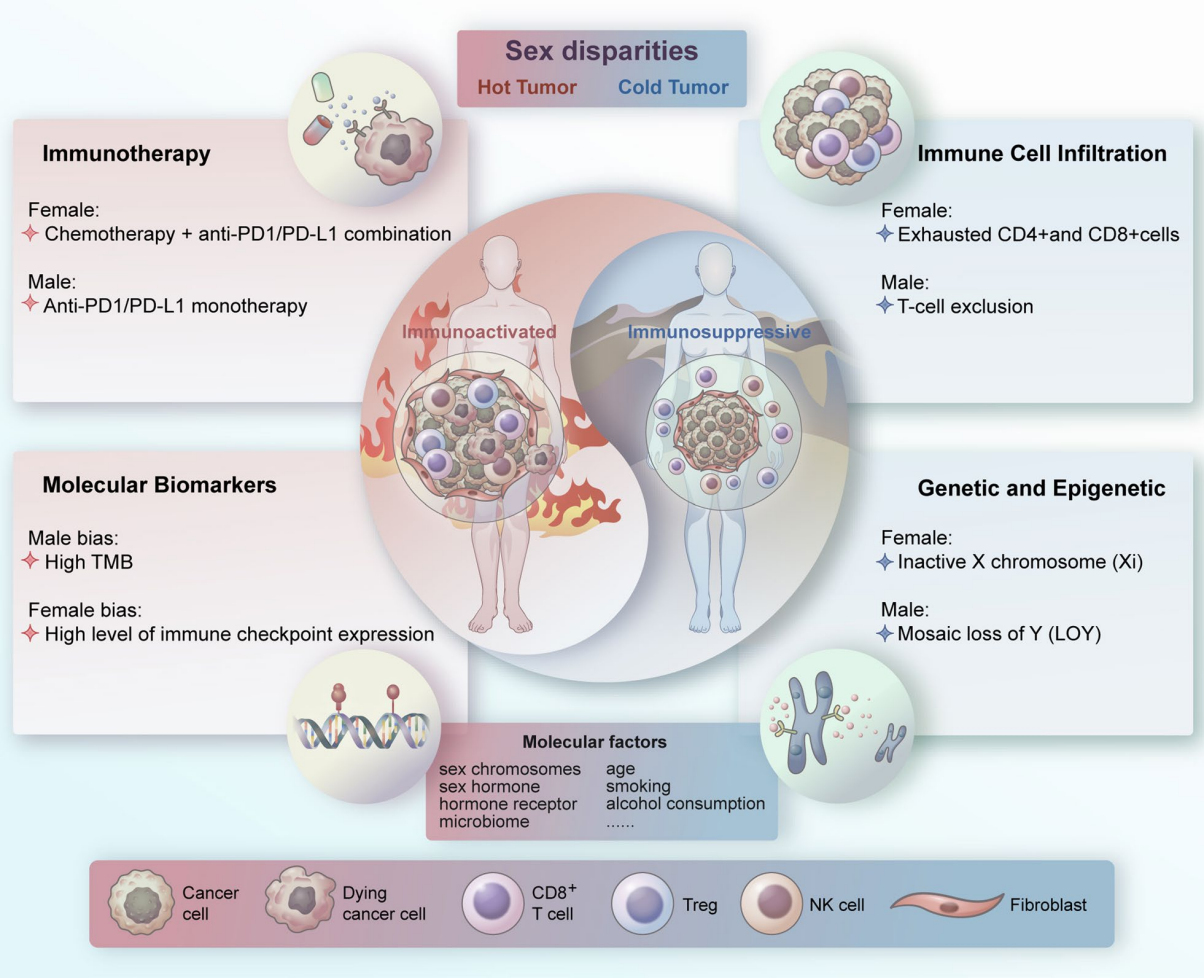
Overview of sex disparities for cancer immunotherapy. Older and male patients are more inclined to hot tumors, also known as immunosuppressive tumors; tumor cells are more visible to the immune system and open to attack when stimulated with ICB. Younger and female patients are more inclined to cold tumors, also known as immune cell infiltrating tumors; tumor cells are more invisible to the immune system and have a poor response to ICB therapy

Enhancing the immune system's response to cancer is core to the new wave of immunotherapies [30], and establishing their efficacies between the sexes is particularly interesting (Fig. 1). Immune checkpoint inhibitors (ICIs) prevent tumor cells from escaping effective T cell-mediated immune surveillance by reenergizing the tumor-specific T cell response [31]. Castro et al. explored why immune checkpoint blockade (ICB) responses in multiple cancer types clinical trials are inferior in young and female patients compared to older and male patients. MHC molecules could not detect tumor driver mutations in the early stage of tumorigenesis. This failure phenomenon is more prevalent in younger and female patients, implying a more decisive immune influence early in the tumorigenic process [31]. This finding is consistent with recent meta-analyses across multiple tumors showing sex- and age-dependent differences in response to ICB [28, 32, 33].

Furthermore, tumors from younger females had the most vital immune selection compared to the other patient cohorts (older females, younger males, and older males), which indicates that sex and age have additive effects [31]. Females and younger individuals are considered less able to elicit an efficient host anti-tumor response, probably because of depletion of immunogenic mutant peptides and consequent non-recognition of cancer by the immune system. This study might account for the ineffectiveness of ICB in this patient population.

In addition, the determination of sex disparities in the ICIs response requires the inclusion of immune and molecular characteristics and the adjustment of confounding factors, including race, smoking status, tumor stage, histological type, tumor purity, and diagnosis age [34]. Unfortunately, cancer heterogeneity hampers definitive conclusions when pooling data across different cancer types and treatments [26, 27]. Moreover, insufficient female subjects often lead to the underrepresentation of females [34].

## Sex disparities in anti-cancer immune response

Harnessing an antitumor immune response has been a fundamental strategy in cancer immunotherapy. Immunity differs between the sexes. Females have more excellent adaptive and innate immune responses than males and confer more excellent antiviral T cell immunity and anti-cancer response [9, 35]. In part, this is attributed to the capacity of low physiological levels of estrogen to stimulate the production of the acute inflammatory cytokines interleukin-6 (IL-6) and tumor necrosis factor (TNF), which are active in infection control. By contrast, higher levels of estrogen dampen the expression of these cytokines. Testosterone also reduces TNF levels. Overall, females are more effective at dealing with infection than men, which corresponds to the cancer risk associated with persistent exposure to chronic infection-related inflammation [9, 36].

The immune system is influenced by genetics and sex hormones, and the interaction between tumor cells, stromal cells, and extracellular molecules within TME. Female cells have shown a more remarkable ability to overcome cellular stress by inducing protective mechanisms, such as autophagy and ferroptosis [37, 38], and more antioxidant defenses than male cells [39].

Immune infiltration was more abundant in women's tumors, and all the T-cell subpopulations were significantly enriched in the TME of women. In contrast, the TME of men has only higher type 2 T-helper cells (Th2-cells) [40]. Healthy young adult females have an activated/mature neutrophil profile characterized by enhanced type I IFN pathway activity, proinflammatory responses, and distinct bioenergetics. These differences between females and males are cell-specific and likely driven by sex hormones [41].

Compared with men, women's tumor microenvironment (TME) was significantly enriched for many innate and adaptive immune cell types, including specific T-cell subpopulations. In females, the TME of NSCLC was characterized by significantly greater T-cell dysfunction status, higher expression of inhibitory immune-checkpoint molecules, and higher abundance of immune-suppressive cells, including CAFs, MDSCs, and Tregs [40]. On the contrary, the TME of NSCLC in males was characterized by a lower abundance of several innate and adaptive immune cell types and a T-cells excluded phenotype. Such poorer immune infiltration of men's tumors could depend on a less efficient tumor neoantigens presentation to the immune system (impaired neoantigens presentation mechanisms)due to lower expression levels of HLA class I and II molecules and higher frequency of HLA type I LOH events. Another mechanism underlying the lower immune infiltration of men's tumors was the higher degree of hypoxia in TME, which has been reported to impair the infiltration and proliferation of immune cells [40].

## Sex disparities in genetic and epigenetic regulations

High-throughput sequencing and genome-wide association studies have revealed a sex bias in human diseases. Cancer is driven mainly by mutational processes, and an overall male bias has been associated with sex differences in DNA repair and genome folding [42, 43]. Broad studies demonstrated a clear sex bias in the mutation burden that is generally higher in male than in female tumors—both overall cancer and several specific tumor types, such as bladder, kidney, liver, and skin cancers [44–46]. The sex bias in the mutation burden of the cancer genomes was associated with decreased gene expression in DNA mismatch repair [44], suggesting that lower efficiency of genome repair mechanisms might underlie sex differences in the frequency of mutations [47, 48]. Moreover, recent studies are showing that two main regulators of genome topology (i.e., CTCF and COHESIN) bind chromatin in a sex-specific manner [49, 50] and that mutations at CTCF binding sites can act as tumor drivers [51]. Sex-specific chromatin conformations might, therefore, affect the efficiency with which DNA damage is repaired, providing a further mechanistic explanation for sex differences in tumor development.

The sex chromosomes play an essential role in the genetic and epigenetic foundation of human sex bias, which has been widely underestimated due to limitations in sequencing their repeat-rich regions. The core of sex differences is sex chromosomes, namely, X and Y allotropes [20]. An inactivated X chromosome results from dosage compensation between males and females [52]. There are fundamental differences in gene expression between typical XY males and XX females. Males inherit their X chromosomes from their mothers and Y chromosomes from their fathers. Females inherit one X chromosome from each parent but only completely express one, termed the active X chromosome (Xa). Long non-coding RNA X-inactive specific transcript (XIST) silences one X of each pair, namely, the inactive X chromosome (Xi), during early development in a random manner.

The unique male Y is pivotal in determining the male sex. Albeit the third smallest chromosome, with approximately 60 protein-coding genes and 100 non-coding RNAs [53], Y encodes functional tumor suppressors [54]. An expanding body of evidence suggests that somatic loss of the sex-determining Y chromosome, referred to as mosaic loss of Y (mLOY), might be an important biomarker for elevated mortality rates among men, perhaps indirectly or through events on the Y chromosome itself [55]. The latest study report that hematopoietic mLOY causally contributes to age-dependent cardiac fibrosis and mortality in men [56]. Y chromosome-deficient cardiac macrophages overactivate a profibrotic signaling network, leading to cardiac fibroblast proliferation and activation, excessive matrix production, and diminished heart function [56]. Haupt et al. found that the Y is enriched with immune-suppressive healing genes, fitting the anti-inflammatory M2-like tumor-associated macrophage signature [36]. M2-like tumor-associated macrophages are related to increased tumor vascularization, tumor immunosuppression, and poor prognosis in response to chemotherapy [57]. Therefore, targeting M2-like male macrophages may effectively strengthen cancer therapy [58].

Sex effects on gene expression are ubiquitous. Yuan et al. found that sex-biased gene expression or DNA methylation signatures were enriched in the sex chromosomes [59]. Whereas X-linked genes with higher expression in females suggest candidates for escape from X-chromosome inactivation, sex-biased expression of autosomal genes suggests hormone-related transcription factor regulation and a role for additional transcription factors, as well as sex-differentiated distribution of epigenetic marks, particularly histone H3 Lys^27^ trimethylation (H3K27me3) [20].

Epigenetic regulation is emerging as a possible dictator of sex dimorphism in healthy males and females [21]. Still, its impact on cancer sex disparity is yet to be fully appreciated [60], warranting further investigation. In analyzing 8,279 sample-specific gene regulatory networks, Lopes-Ramos et al. found significant sex differences in gene regulation across all 29 tissues analyzed. Transcription factors (T.F.s) display sex-biased targeting patterns, which uncovered that they were associated with tissue-specific functions and diseases[21]. The immunosuppressive microenvironment is driven by CD4 + regulatory T (Treg) cells, a significant obstacle to successful tumor immunotherapy [61]. Treg characteristically expresses the X-linked FOXP3 [62], a transcriptional regulator crucial to Treg cell development and function. The X-linked Toll-like receptor 8 (TLR8) also performs a central role in Treg cell biology. Activation of TLR8 in Treg cells impairs immunosuppression. Targeting Treg cells relieved the inhibition of effector T cells [61]. TLR8 agonist, new cancer immunotherapy, is expected to become potent anti-cancer agents in males [63].

X-linked genes endow genders with different anti-cancer capacities. For instance, TLR7 and TLR8 coding genes are tandem duplicates on the X chromosome [64], and essential details about their transcriptional regulation differences between the sexes are emerging. The expression of TLR7 in human female B cells and myeloid cells has been proven to be associated with gender differences [65]. As for mice, X-linked TLR7 is also more highly expressed in these cells of males than females; and this is attributed to lower methylation levels across the maternal X-chromosome. Females, in contrast, either express their maternal less methylated copy or their paternal more highly methylated X, showing a global lower level of gene expression than males [66]. Notably, although in humans, the level of CD4 + T cells decreases with age, the number of circulating CD4 + T cells in females is higher than that in age-matched males; there are higher numbers of CD4 + T cells circulating in females than in age-matched males, even as levels decline with aging [9, 67].

The tumor suppressor gene TP53 is the most frequently mutated gene in cancer. Wild-type p53 can suppress tumor development by multiple pathways [68]. Loss of the tumor suppressor p53 causes stochastic Xi reactivation and biallelic expression of X-linked genes [69]. This, together with recent evidence of genetic interactions between p53 and the X chromosome across 12 distinct cancer types, suggests that p53 might play a role in the sex bias of cancers by increasing the probability of stochastic transcriptional events on the Xi [69]. As one of the genes transcriptionally regulated by p53, TLR8 expression level also showed significant sex disparity. This transcriptional regulation level is controlled by a single nucleotide polymorphism (SNP) in the human TLR8 promoter. Female X chromosome chimerism seems to affect this single nucleotide polymorphism and thus affects TLR8 expression [70].

Yuan et al. performed a multidimensional analysis of molecular differences between male and female patients and revealed a two-group molecular classification of cancer types (weak sex-effect group vs. strong sex-effect group). They found that > 50% of clinically actionable genes show sex-biased signatures in some tumor types, suggesting a need for sex-specific therapeutic strategies [59]. In addition, Yuan et al. identified 11 sex-biased genes in lung adenocarcinoma (LUAD); one of the most notable genes is STK11 (also known as LKB1), which encodes a major upstream kinase that activates the energy-sensing AMPK pathway and is frequently mutated in a variety of cancers [71]. Their result suggested that sexual dimorphism might play a pivotal role in reprogramming pathways that mediate the cellular metabolism of cancer [72, 73]. KRAS is the most common oncogenic driver in LUAC, and STK11 alterations have been shown to confer primary resistance to PD-1 axis blockade in KRAS mutant lung adenocarcinomas [74]. STK11 and KEAP1 mutations (STK11 mutant [STK11^MUT^] and KEAP1^MUT^) are among the most often mutated genes in LUAD. Recent studies have shown that STK11 and KEAP1 mutations confer worse outcomes to immunotherapy among patients with KRAS^MUT^ but not among KRAS wild-type (KRAS^WT^) LUAD. Tumors harboring concurrent KRAS/STK11 and KRAS/KEAP1 mutations display distinct immune profiles regarding gene expression and immune cell infiltration [75]. STK11 has been proposed to induce T-cell exhaustion and immunosuppressed or "cold" tumor microenvironment with lower PD-L1 expression [76]. The above may partly explain the poor response of female patients to immunotherapy.

## Sex disparities in sex hormone and hormone receptor-mediated anti-tumor immunity

Sex hormones could modulate the interaction between genes and the immune response [77]: progesterone has extensive anti-inflammatory effects; androgens suppress the activity of immune cells; estradiol improves cell-mediated and humoral immune responses. Much information exists on the impact of sex steroid hormones on cancer development in non‑reproductive organs. These lipid-soluble hormones can enter the plasma membrane of target cells and interact directly with intracellular receptors that can shuttle to the nucleus to affect gene expression [78]. The action of these hormones extends, at the epigenetic level, to DNA methylation and chromatin conformation [79, 80].

Sex hormone signaling pathways are likely to affect cancer susceptibility and prognosis through multiple intrinsic and extrinsic mechanisms. The latest study by Vellano et al. revealed that the expression of androgen receptors was significantly increased in male patients during BRAF/MEK targeted therapy and was associated with therapeutic resistance [81]. Yang et al. identify that AR signaling accelerates the transition from stem cell-like CD8 + T cells to terminally exhausted CD8 + T cells in males, leading to sex-biased anti-tumor immunity. In contrast, AR signaling inhibition reprograms CD8 + T cells into a stem cell-like state to potentiate cancer immunotherapy [82]. Male CD8 + T cells exhibited impaired effector and stem cell-like properties compared with female CD8 + T cells. Mechanistically, androgen receptors can inhibit the activity and stemness of male tumor-infiltrating CD8 + T cells by regulating epigenetic and transcriptional differentiation programs [82]. This study reveals the sexual differences in CD8 + T cell stemness programs in the TME and highlights a sex-based therapeutic target for cancer immunotherapy.

Androgen significantly contributes to sex-related differences in various diseases by regulating inflammatory environments [9]. Since androgen receptor (AR) is expressed in both immune and stromal cells, the involvement of AR has been shown to cause sex differences in immune responses via an immune-cell-intrinsic or immune-cell-extrinsic pattern [83–85]. Guan et al. demonstrate that androgen receptor (AR) blockade sensitizes tumor-bearing hosts to effective checkpoint blockade by directly enhancing CD8 T cell function. Inhibition of AR activity in CD8 T cells prevented T cell exhaustion and improved responsiveness to PD-1-targeted therapy via increased IFNγ expression [86]. Kwon et al. investigated differences in tumor immune responses between males and females in non-reproductive organ cancers; it revealed the promoting role of androgen-mediated CD8 + T cell dysfunction in cancer and a pertinent contribution from AR as a direct transcriptional trans-activator of Tcf7/TCF1 [22]. Moreover, ablation of the androgen–AR axis rewires the tumor microenvironment to favor effector T cell differentiation and potentiates the efficacy of anti-PD-1 immune checkpoint blockade [22]. In conclusion, AR-mediated predisposition for CD8 + T cell exhaustion interferes with eliminating nascent immunogenic malignant cells, leading to a male bias in cancer incidence and mortality.

## Sex disparities in molecular biomarkers of immunotherapy

Immunotherapy, represented by immune checkpoint inhibitors (ICIs), transforms cancer treatment. However, only a fraction of patients respond to ICI, and there is an unmet need for biomarkers to identify patients more likely to respond to ICI [87]. A recent study has found sex-based differences in molecular biomarkers and immune checkpoint expression in multiple tumor types treated with ICB [34]. In 2020, Ye et al. performed a pan-cancer analysis that reported heterogeneity in molecular biomarkers between the sexes, including tumor mutation burden (TMB), individual gene mutation (PBRM1, BRCA2), T cell-inflamed gene expression profile (GEP), neoantigen load, cytolytic activity (CYT) and protein expression of checkpoints (CTLA-4, PD-L1, PD-L2). A divergent pattern of sex-associated differences across different cancer types (Table 1), such as the male bias in melanoma, has a high tumor mutational burden [34, 45, 88]. In contrast, the female bias in non-small cell lung cancer has a high level of immune checkpoint expression [34].

**Table 1:** Summary of sex disparities in molecular biomarkers of immunotherapy

Biomarker	Cancer type	Tissue type for biomarker assessment	Sex bias
TMB	Multiple cancer types	Blood or tumor tissue	Male bias
Neoantigen load	Melanoma and NSCLC	Blood or tumor tissue	TBD
T cell-inflamed GEP	Multiple cancer types	Tumor tissue	TBD
CYT	Multiple cancer types	Tumor tissue	TBD
PD-L1 expression	Multiple cancer types	Tumor tissue	Female bias
HLA class I diversity	Melanoma and NSCLC	Blood	TBD
LOH at HLA class I alleles	Melanoma	Tumor tissue	TBD
TCR clonality change	Melanoma	Blood or tumor tissue	TBD
CNV	Multiple cancer types	Tumor tissue	TBD

*TMB* tumor mutational burden, *NSCLC* non-small-cell lung cancer, *HLA* human leukocyte antigen, *LOH* loss of heterozygosity, *TCR* T cell receptor, *GEP* gene expression profile, *CYT* cytolytic activity, *CNV* copy number variation, *TBD* to be determined

We know that tremendous efforts have been undertaken to identify biomarkers to predict the response to immunotherapy. Tumors with a high tumor mutation burden (TMB) [89–91] tend to present more immunogenic neoantigens to enhance the ability of T cells to recognize and kill tumor cells [92]. Female tumors tend to have a lower tumor mutational burden (TMB) due to strong MHC class II-based immune selection during tumor development [31]. This weaker antigenicity of female tumors may lead to a less effective anti-tumor immune response upon ICI treatment in female patients.

The study of Wang et al. showed that the performance of TMB in ICI response prediction for female patients is significantly better than for male NSCLC patients [93]. Using two independent cohorts of, respectively, advanced NSCLC patients treated with anti-PD1/anti-PDL1 drugs, the analysis by Conforti et al. suggested that TMB could have a solid and linear association with both PFS and OS only in women and that considering different TMB cutoff points in both genders may improve its predictive value for both. It is important to note that they analyzed only data from patients treated with anti-PD1/PD-L1 drugs as monotherapy. Therefore, the results could not be valid for other immunotherapy strategies, including anti-CTLA4 drugs given alone or combined with anti-PD1/PD-L1 antibodies or combining chemotherapy with ICIs [40].

PD-L1 is actively expressed on both tumor cells and antigen-presenting cells, and inhibition of PD-1 potentially affects multiple steps in the early stage of the lymph node and subsequent immune response in the tumor microenvironment [94]. PD-L1 expression is another independent biomarker for predicting ICI response, even though its performance is problematic [95]. The study of Wang et al. showed that the predictive power of PD-L1 expression on ICI response is not affected by the patients' sex [93]. Probably because PD-L1 expression itself is directly involved in ICI function, the predictive power of PD-L1 expression is not affected by sex differences.

Telomerase reverse transcriptase (TERT) mutations lead to aberrantly upregulating TERT expression and ultimately enable telomere maintenance, which achieves the unlimited proliferative capacity of tumor cells [96]. Moreover, TERT mutations are biomarkers of tumor aggressiveness and poor prognosis in several human cancer types [97, 98]. Lately, Li et al. identified that TERT mutation might serve as a sex-specific cancer biomarker, and the TERT mutation frequency of melanoma was higher in male patients. They found that male patients with TERT mutation may be more likely to benefit from immunotherapy [99].

Presently, dozens of other biomarkers are being studied, which may involve different biomarkers for different cancers and different stages of treatment. Combining chemo-immunotherapies or immuno-immunotherapies is one of the most effective ways of treating cancer. Biomarkers may not only help patients treated with checkpoint inhibitors to choose monotherapy but also immunotherapy combinations. Some cancers may respond better to combination therapy. In addition, it is meaningful to screen out these patients. It seems inevitable to use a combination of biomarkers to predict efficacy, and sex may be one of the critical variables.

The concentrations of biomarkers present in patients may depend on the sex of the patient [35]. Ramsey et al. analyzed the levels of more than 170 proteins and small-molecule biomarkers in men and women with different hormonal states. They reported that 56% of biomarkers concentration varied between men and women [100]. However, the sex difference in the predictive power of biomarkers is highly intriguing. The sex difference in the predictive power of biomarkers in cancer immunotherapy has rarely been accurately reported; thus, this finding points to a new field of research. Perhaps in the future, we should consider analyzing the predictive ability of cancer immunotherapy biomarkers based on the sex of the subjects.

## Sex disparities in immune-related adverse events (irAEs)

Decisions regarding immunotherapy use or whether a combination approach is warranted should be guided by rational, mechanistic insight to maximize disease control, reduce side effects, and minimize cost. Furthermore, although immunotherapies have fewer adverse effects overall than chemotherapy [101, 102], it is crucial to identify patients most at risk of therapy-related toxicity to be appropriately monitored and treated. Cancer immunotherapies have changed the cancer treatment landscape during the past few decades. Among them, immune checkpoint inhibitors, which target PD-1, PD-L1, and CTLA-4, are increasingly used for certain cancers; however, this increased use has resulted in increased reports of immune-related adverse events (irAEs). Unlike traditional cancer therapies, irAEs typically have a delayed onset and a prolonged duration and may involve any organ or system [103–105]. Thoughtful management of irAEs is essential in optimizing the quality of life and long-term outcomes.

Sex differences in irAEs in cancer treatment are not known. In 2021, Jing et al. took advantage of different analytical approaches with multi-source data, combining the benefits of the three data sources from clinical, real-world pharmacovigilance, and omics data; analyzed sex-associated differences in irAEs among cancer patients. They observed minimal sex-associated differences in irAEs among cancer patients who received immune checkpoint inhibitor therapy. It may be unnecessary to consider sex effects on irAE management in clinical practice [106]. However, in 2022, Unger et al. analyzed treatment-related adverse events (AEs) by sex in SWOG phase II and III clinical trials conducted between 1980 and 2019, excluding sex-specific cancers. They indicate that the risk of cardiotoxicity among nonsex-specific cancers may be higher for women than men, especially for those treated with chemotherapy or immunotherapy [107]. Sex may independently modulate drug toxicity, including for novel treatments.

## Perspectives and significance

Sex disparity in cancer is undeniable and emerging with complex interplaying elements. Inherent genetic differences, overlapping epigenetic alterations, and sex hormone influences underpin everything. Relevant to the new wave of precision cancer medicine is the sex-based molecular signatures associated with more than 50% of genes identified as clinically actionable as therapeutic targets or biomarkers [59].

Curing cancer through precision medicine is the paramount aim of the new wave of molecular and genomic therapies. In efforts to understand the molecular basis of sex differences in disease and other phenotypes, it is essential to note that the connection between the molecular changes observed and complex phenotypes is likely to be complicated by many compensatory and buffering effects [108]. Despite extensive sex differences at the transcriptome level, most biology at all phenotype levels is shared between males and females. The sex differences in immunotherapy are based on snapshots of older individuals with advanced cancer. Sex is highly related to many behavioral characteristics and external environments, such as smoking [109] and alcohol consumption [15], and eliminating sex differences caused by inherent biological and gender environments is a more critical challenge. Ideally, cancer therapy aims to maximize treatment efficacy while limiting toxicity. In addition, identifying and developing predictive biomarkers of ICI response for sex bias, both to enable a precision medicine approach in cancer immunotherapy and to better understand and overcome resistance mechanisms.

## Data Availability

Not applicable.

## Abbreviations

ICIs: Immune checkpoint inhibitors
ICB: Immune checkpoint blockade
PD-1: Programmed cell death 1
PD-L1: Programmed cell death-ligand 1
CTLA-4: Cytotoxic T-lymphocyte antigen-4
NSCLC: Non-small cell lung cancer
LUAD: Lung adenocarcinoma
IL-6: Interleukin-6
TNF: Tumor necrosis factor
TME: Tumor microenvironment
CAFs: Cancer-associated fibroblasts
MDSCs: Myeloid-derived suppressor cells
Tregs: Regulatory T-cells
LOH: Loss of heterozygosity
AR: Androgen receptor
TFs: Transcription factors
TMB: Tumor mutational burden
GEP: Gene expression profile
CYT: Cytolytic activity
irAEs: Immune-related adverse events
